# Small Intestine Neuromuscular Dysfunctions and Neurogliopathy in a Mouse Model of High-Fat Diet-Induced Obesity: Involvement of Toll-Like Receptor 4

**DOI:** 10.3390/ijms262110710

**Published:** 2025-11-03

**Authors:** Sofia Faggin, Silvia Cerantola, Annalisa Bosi, Cristina Giaroni, Eleonora Napoli, Edoardo Vincenzo Savarino, Martina Contran, Andrea Porzionato, Carolina Pellegrini, Luca Antonioli, Valentina Caputi, Maria Cecilia Giron

**Affiliations:** 1 Department of Pharmaceutical and Pharmacological Sciences, University of Padova, 35131 Padova, Italy; sofia.faggin@unipd.it (S.F.); silvia.cerantola.2@gmail.com (S.C.); 2 Department of Medicine and Surgery, University of Insubria, 21100 Varese, Italy; annalisa.bosi@uninsubria.it (A.B.); cristina.giaroni@uninsubria.it (C.G.); 3 Department of Neurology, School of Medicine, University of California Davis, Sacramento, CA 95817, USA; enapoli@ucdavis.edu; 4 Department of Surgery, Oncology and Gastroenterology, University of Padova, 35131 Padova, Italy; edoardo.savarino@unipd.it; 5 Section of Human Anatomy, Department of Neuroscience, University of Padova, 35131 Padova, Italy; martina.contran@unipd.it (M.C.); andrea.porzionato@unipd.it (A.P.); 6 Department of Clinical and Experimental Medicine, University of Pisa, 56126 Pisa, Italy; carolina.pellegrini@unipi.it (C.P.); luca.antonioli@unipi.it (L.A.); 7 Department of Poultry Science, University of Arkansas, Fayetteville, AR 72701, USA

**Keywords:** obesity, Toll-like receptor 4, high-fat diet, enteric nervous system, serotonin, inflammation, neurodegeneration

## Abstract

Obesity is associated with enteric dysfunctions, including gut dysmotility and neurodegeneration, which may involve Toll-like receptor 4 (TLR4) signaling. To investigate this relationship, we examined the impact of TLR4 deficiency on the enteric nervous system (ENS) of the small intestine in a mouse model of high-fat diet (HFD)-induced obesity. Male TLR4^−/−^ and wild-type (WT) C57BL/6J mice were fed either a standard diet (SD; 18% kcal fat) or an HFD (60% kcal fat) for 8 weeks. ENS alterations were evaluated using real-time qPCR and confocal immunofluorescence microscopy on longitudinal muscle–myenteric plexus (LMMP) whole-mount preparations. Alterations in gut motility were evaluated by assessing stool frequency, transit of a fluorescent-labeled marker, and isometric motor responses of ileal preparations to receptor- and non-receptor-mediated stimuli. In WT mice, HFD induced delayed gastrointestinal transit, impaired cholinergic and nitrergic responses, and altered 5-HT-mediated concentration–response curves. These functional deficits were accompanied by neuroglial network disruption, myenteric neurodegeneration, loss of ChAT^+^ and nNOS^+^ neurons, and increased 5-HT ileal tissue levels. In contrast, TLR4 deficiency mitigated body weight gain and largely prevented HFD-induced structural and functional alterations. Overall, our findings highlight a key role for TLR4 signaling in modulating small intestine inflammation and ENS remodeling associated with obesity.

## 1. Introduction

High-fat diet (HFD) is a well-established contributor to the development of overweight or obesity, conditions that currently affect over one-third of the global population and are projected to exceed 50% by 2030 [1]. Obesity, a complex multifactorial disorder arising from an imbalance between energy intake and expenditure, is characterized by chronic, low-grade inflammation affecting multiple tissues, including the gastrointestinal (GI) tract [2,3]. Adipocytes and macrophages within adipose tissue are major sources of pro-inflammatory mediators, such as interleukins, tumor necrosis factor (TNF), and monocyte chemoattractant protein 1 (MCP-1) [4]. This persistent inflammatory state plays a central role in the development of various obesity-related comorbidities, including type 2 diabetes mellitus, cardiovascular diseases, cognitive decline, dementia, and certain cancers [5,6].

Emerging clinical data indicate a strong association between obesity and chronic GI disturbances, such as gastroesophageal reflux, diarrhea, and constipation, which further compromise patients’ quality of life [7,8]. Preclinical studies demonstrate that chronic HFD exposure increases intestinal permeability and gut inflammation, leading to enteric nervous system (ENS) impairment and disrupted gut motility [2,7,9,10]. Enhanced permeability facilitates the translocation of bacterial endotoxins, driving low-grade inflammation and metabolic dysfunction [11]. HFD also alters gut microbiota composition, typically reflected by an increased Firmicutes-to-Bacteroidetes ratio, a dysbiotic profile linked to greater energy harvest and obesity development [12,13]. Moreover, HFD has been reported to modify intestinal transit time, with some studies describing accelerated colon transit [14] while others report delayed transit [13], suggesting that outcomes depend on diet composition and duration [14].

Toll-like receptors (TLRs) represent a class of pattern-recognition receptor family that recognizes pathogen- and damage-associated molecular patterns and activates innate immune responses. Among TLRs, TLR4 induces innate immune responses when stimulated by lipopolysaccharides (LPS), the main component of the outer membrane of Gram-negative bacteria [15,16]. Human polymorphisms for TLR4 genes have been associated with increased susceptibility to infectious and non-infectious diseases (i.e., inflammatory bowel disease, metabolic syndrome, obesity, T2D, cardiovascular disorder, and cancer) [17,18,19]. Polymorphisms within the coding or promoter regions of the TLR4 gene can modify the receptor’s responsiveness to lipopolysaccharides (LPS), enhancing immune and inflammatory signaling and thereby contributing to the development of obesity and related metabolic disorders. Beyond its immunologic role, TLR4 signaling also regulates ENS homeostasis and GI function [16,20]. Under physiological conditions, TLR4^−/−^ mice display altered gut motility and changes in ENS neuronal composition in both the small intestine and colon [16,20,21,22,23], yet they are protected from obesity-induced inflammation and insulin resistance [24,25,26]. Moreover, HFD exposure does not significantly affect GI transit or colonic myenteric cell density in TLR4-deficient mice, underscoring the critical role of TLR4 signaling in enteric neuroinflammation and motility disturbances in diet-induced obesity [13,24,25,27].

Despite recent progress, the impact of HFD on small intestinal contractility, neurotransmission pathways, and integrity of the myenteric nervous system in the absence of TLR4 signaling remains poorly understood. Clarifying how TLR4 modulates ENS circuitry in response to dietary fat could deepen our understanding of the mechanisms linking diet, immune signaling, and GI motility. To address this, in the present study we examined the effect of short-term 8-week HFD feeding in WT and TLR4^−/−^ mice. Our findings show that HFD induced delayed GI transit, dysfunction of excitatory cholinergic and inhibitory nitrergic neurotransmission, and altered serotonergic signaling and metabolism, effects that were largely prevented by TLR4 deficiency.

## 2. Results

### 2.1. Influence of TLR4 Signaling on HFD-Induced Obese Phenotype

**Figure 1:**
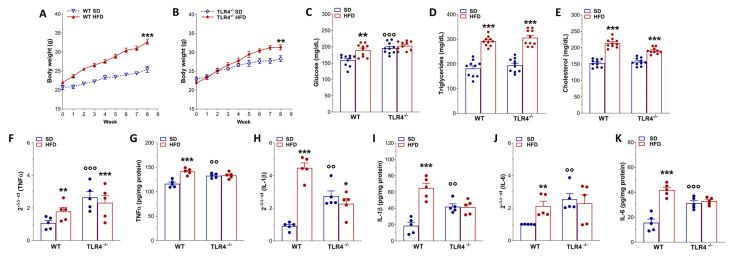
Phenotypic changes and inflammation marker expression after HFD-induced obesity. (**A**,**B**) Body weight gain in WT (**A**) and TLR4^−/−^ (**B**) mice after 8 weeks of HFD treatment; *n* = 10 mice/group. (**C**–**E**) Glucose (**C**), triglycerides (**D**), cholesterol (**E**) plasma levels in WT and TLR4^−/−^ mice fed with SD or HFD; *n* = 10 mice/group. (**F**,**H**,**J**) Real-time qPCR analysis of TNFα (**F**), IL-1β (**H**), and IL-6 (**J**) mRNA levels in ileal tissues from WT and TLR4^−/−^ mice fed with SD or HFD; *n* = 5 mice/group. (**G,I,K**) ELISA analysis of TNFα (**G**), IL-1β (**I**), and IL-6 (**K**) protein levels in ileal tissues from WT and TLR4^−/−^ mice fed with SD or HFD; *n* = 5 mice/group. * *p* < 0.05, ** *p* < 0.01, *** *p* < 0.001 vs. related SD genotype; °° *p* < 0.01, °°° *p* < 0.001 vs. WT SD mice.

To verify that HFD feeding reproduced the hallmark features of obesity, we monitored body weight gain, blood metabolic parameters, and ileal inflammatory mediator levels in both genotypes under HFD and SD conditions. After 8 weeks, HFD produced significant body weight gain in both WT and TLR4^−/−^ mice (+28% and +10%, respectively; Figure 1A,B) compared to SD-fed mice. Interestingly, in TLR4^−/−^ mice the body weight increase occurred at week 5 of HFD feeding (Figure 1A,B and Supplementary Figure S1), whereas in WT mice this increase was observed from the beginning of HFD exposure. Higher plasma levels of glucose, triglycerides, and cholesterol (+20%, +48%, and +40%, respectively; Figure 1C,D,E) were found in WT HFD mice compared to the SD group, as previously reported [28]. In TLR4^−/−^ mice, HFD induced a marked increase in triglycerides and cholesterol (+58% and +25%, respectively; Figure 1D,E) without altering glucose levels (Figure 1C).

To investigate whether HFD consumption might determine gut inflammation, we measured pro-inflammatory gene expression in small intestinal mucosa-deprived tissue from 8-week-old SD- and HFD-fed mice of both the genotypes. We found a marked increase in TNFα, IL-1β, and IL-6 mRNA and protein levels (Figure 1F,H,J) in WT HFD mice compared to related SD animals, indicating the presence of an inflammatory state in the small intestine neuromuscular compartment, as observed previously in the colon of HFD-fed mice [13,24]. Under SD conditions, TLR4^−/−^ mice exhibited high pro-inflammatory cytokine levels compared to WT SD mice, which, surprisingly, were not affected by 8-week HFD (Figure 1F–K).

### 2.2. TLR4 Signaling Is Involved in HFD-Induced Gastrointestinal Dysmotility

**Figure 2:**
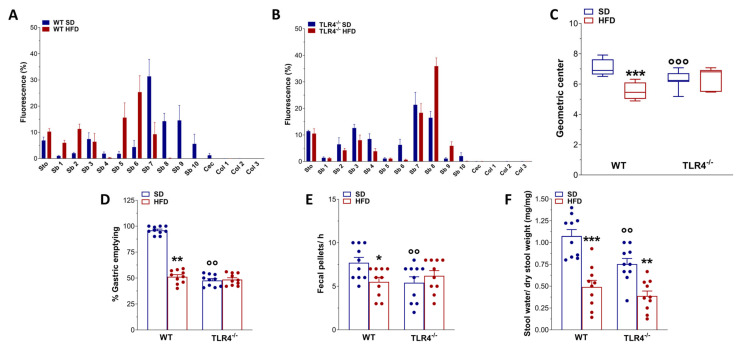
TLR4 signaling influenced HFD-effects on gastrointestinal motility. (**A**,**B**) Distribution of the non-absorbable FITC dextran (70 kDa), expressed as % fluorescence ± SEM across the different segments of the gastrointestinal tract (Sto, stomach; Sb1–Sb10, small intestine segments; Cec, caecum; Col1–Col3, colon segments) in WT (**A**) and TLR4^−/−^ (**B**) mice fed either a standard diet (SD) or a high-fat diet (HFD). (**C**) Geometric center derived from GI transit analysis, reported as median and percentiles. (**D**–**F**) Gastric emptying (**D**), fecal pellet output during a 1 h collection period (**E**), and stool water content (**F**) in WT and TLR4^−/−^ mice fed SD or HFD. Data are reported as mean ± SEM. * *p* < 0.05, ** *p* < 0.01, *** *p* < 0.001 vs. corresponding SD genotype; °° *p* < 0.01, °°° *p* < 0.001 vs. WT SD mice. *n* = 10 mice/group for all tested outcomes (**A**–**F**).

**Figure 3:**
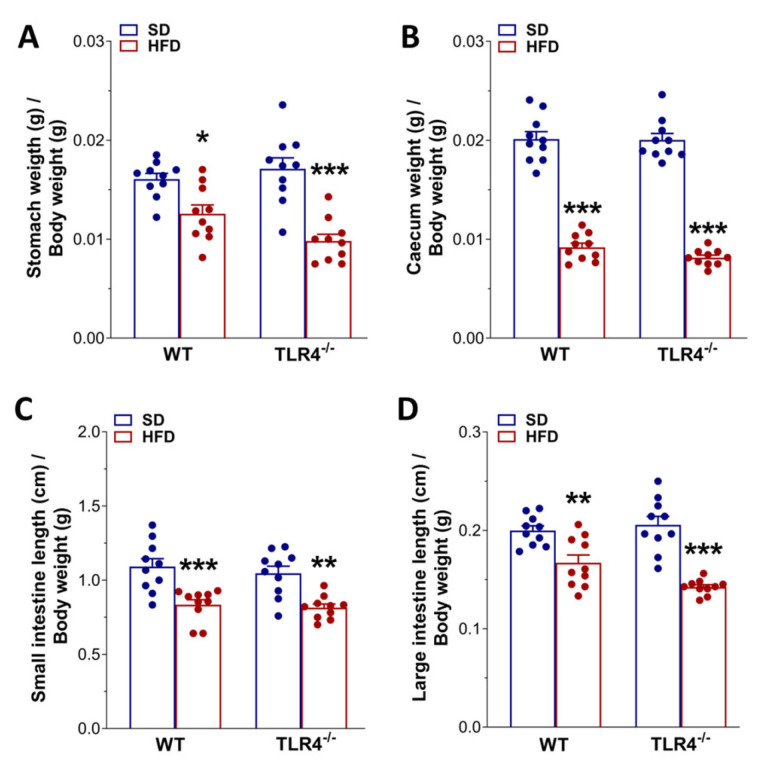
HFD affected gastrointestinal tract morphology. Changes in stomach (**A**) and cecum (**B**) weights, as well as in small (**C**) and large intestine (**D**) lengths normalized to body weight of WT and TLR4^−/−^ mice at the end of the treatment with SD or HFD. Data are reported as mean ± SEM. * *p* < 0.05, ** *p* < 0.01, *** *p* < 0.001 vs. related SD genotype; *n* = 20 mice/group.

In animal models of HFD, obesity contributes to the development of constipation, as generally observed also in obese patients [7]. Therefore, we evaluated GI transit, fecal pellet output, and related water content to uncover any changes in the entire enteric motor propulsive activity and barrier function. Previous findings from our group [16] showed that TLR4^−/−^ SD mice displayed a significant decrease in the geometric center (GC) compared to WT mice (GC_WT SD_ = 6.9 vs. GC_TLR4−/− SD_ = 6.2; Figure 2A–C) associated with a delayed gastric emptying, decreased fecal pellets/hour, and stool water content (Figure 2D–F). However, only in WT mice did HFD prolong the GI transit time, as shown by the marked reduction in the GC compared to SD-fed mice (GC_WT SD_ = 6.9 vs. GC_WT HFD_ = 5.5; Figure 2A,C). Furthermore, reduced gastric emptying, stool expulsion frequency, and fecal water content were also observed in WT HFD mice (Figure 2D–F). In both genotypes, HFD significantly impaired stomach and caecum weight (Figure 3A,B), as well as small intestine and colon length (Figure 3C,D).

### 2.3. TLR4 Signaling Influences Small Intestine Excitatory Cholinergic Neurotransmission Following 8-Week HFD

**Figure 4:**
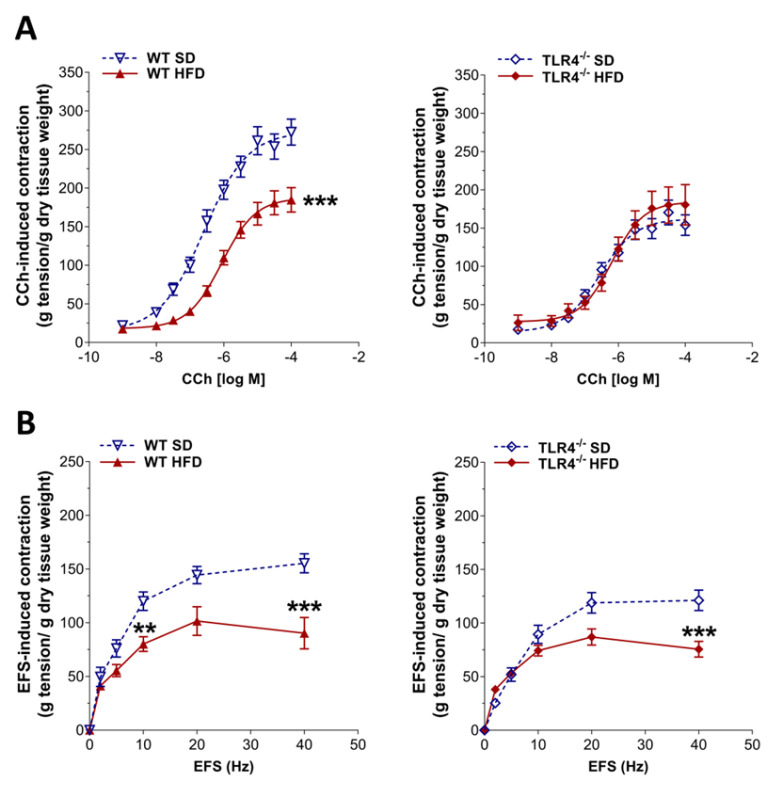
HFD impaired the enteric cholinergic excitatory response in WT mice. (**A**) Concentration–response curves to carbachol (CCh; 0.001–100 µM) and (**B**) neuronal excitatory response induced by EFS (0–40 Hz) in ileal preparations from WT and TLR4^−/−^ mice fed SD or HFD. Data are reported as mean ± SEM. ** *p* < 0.01, *** *p* < 0.001 vs. related SD genotype. *n* = 10 mice/group.

Since HFD exposure delayed the GI transit, we evaluated the excitatory neuromuscular function of isolated ileal preparations from WT and TLR4^−/−^ mice, by analyzing the cumulative dose–response to the non-selective cholinergic receptor agonist CCh. After 8 weeks of HFD, WT ileal segments exhibited a pronounced decrease in CCh-induced contractility in WT mice, evidenced by a downward shift in the concentration-response curve and a 32% reduction in Emax compared with controls (Figure 4A). As previously reported by our group [16], TLR4^−/−^ SD mice displayed a lower CCh-mediated contraction compared to WT animals (Figure 4A), which was not altered following HFD treatment. HFD-induced obesity resulted in a marked attenuation of EFS-evoked contractile responses in both genotypes, with maximal contractions at 40 Hz decreased by 42% in WT and 34% in TLR4^−/−^ (Figure 4B).

**Figure 5:**
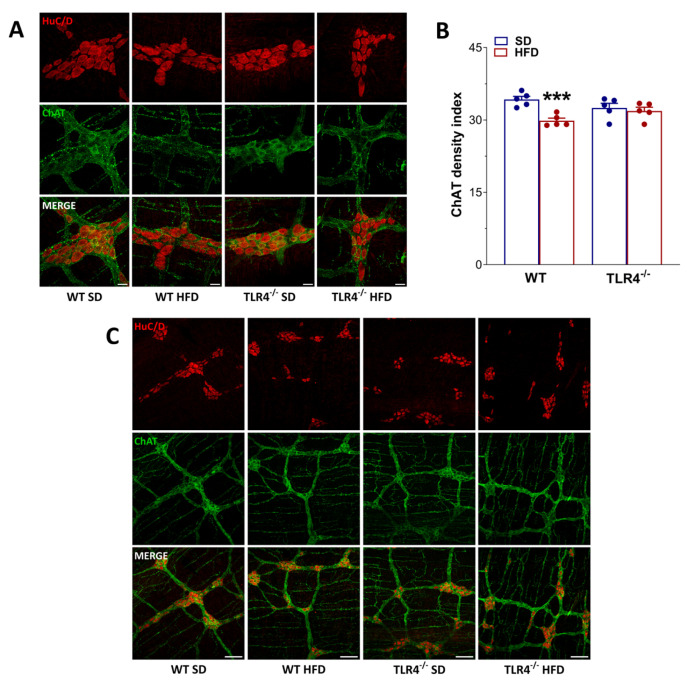
HFD affected the myenteric cholinergic network in WT mice. (**A**,**C**) Representative confocal microphotographs [magnification 63× (**A**) or 20×] (**C**) showing the distribution of ChAT (green, marker for cholinergic neurons) and HuC/D (red, pan-neuronal marker) in LMMP preparations from WT and TLR4^−/−^ mice fed with SD or HFD; scale bars = 20 μm (**A**) or 100 μm (**C**,**B**) ChAT density index in LMMP preparations of WT and TLR4^−/−^ mice fed with SD or HFD. Data are reported as mean ± SEM. *** *p* < 0.001 vs. related SD genotype. *n* = 5 mice/group.

In the mouse ileum, EFS-evoked contractions at frequencies up to 10 Hz are mediated by neuronal cholinergic pathways, evidenced by their sensitivity to both TTX and atropine [29]. Intriguingly, EFS at 10 Hz produced a 33% reduction in cholinergic contraction in ileal segments of WT HFD-fed animals relative to those on SD (Figure 4B), indicative of alterations in enteric cholinergic neurotransmission. Therefore, we evaluated the immunoreactivity of the enzyme ChAT in the myenteric plexus of WT and TLR4^−/−^ mice (Figure 5), to assess the status of the ileal cholinergic network. HFD administration caused a significant reduction in ChAT immunofluorescence (−14%) and disrupted the architecture of the myenteric cholinergic network (Figure 5) only in WT mice compared to WT SD mice, without any alterations in the TLR4^−/−^ group, confirming the involvement of TLR4 signaling in the impairment of cholinergic neuromuscular response.

### 2.4. TLR4 Signaling Affects Ileal Inhibitory Nitrergic Neurotransmission During HFD

**Figure 6:**
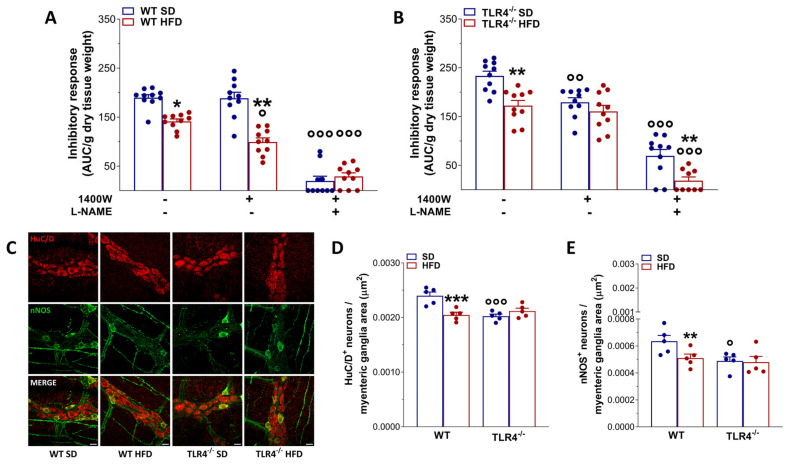
TLR4 signaling influenced HFD-induced enteric neurodegeneration, modulating also NO-mediated relaxation. (**A**,**B**) 10 Hz EFS-evoked relaxation in NANC conditions with or without 10 μM 1400 W (iNOS inhibitor) or 100 μM L-NAME (pan-NOS inhibitor) in ileal segments of WT (**A**) and TLR4^−/−^ (**B**) mice fed with SD or HFD; *n* = 10 mice/group. * *p* < 0.05, ** *p* < 0.01 vs. related SD genotype; ° *p* < 0.05, °° *p* < 0.01, °°° *p* < 0.001 vs. respective control in NANC conditions. (**C**) Representative confocal microphotographs showing the distribution of HuC/D (red) and nNOS (green) and (**D**,**E**) analysis of HuC/D^+^ and nNOS^+^ neurons in ileal LMMPs of WT and TLR4^−/−^ mice fed with SD or HFD (scale bars = 20 μm). Data are reported as mean ± SEM. * *p* < 0.05, ** *p* < 0.01, *** *p* < 0.001 vs. related SD genotype; ° *p* < 0.05, °°° *p* < 0.001 vs. WT SD mice.

Considering that the reduced excitatory contraction could stem from an increased inhibitory tone, we assessed ileal nitrergic neurotransmission. As previously shown [16], in NANC conditions EFS at 10 Hz led to a 1.3-fold increase in the relaxation sensitive to the iNOS inhibitor 1400W and to the pan-NOS inhibitor L-NAME in TLR4^−/−^ mice preparations, whereas in WT segments, EFS-evoked NANC relaxation was almost completely blocked by L-NAME (Figure 6A,B). Following the HFD regimen, WT mice exhibited a significant reduction in the inhibitory response compared to WT mice fed an SD (−26 ± 1%; Figure 6A). Pretreatment with 1400W produced a moderate, non-significant decrease in NANC-mediated relaxation (Figure 6A), whereas L-NAME pretreatment almost completely abolished EFS-evoked NANC relaxation in WT mice on either diet (Figure 6A). On the other hand, in TLR4^−/−^ mice, 8 weeks of HFD also led to a significant attenuation of the inhibitory response relative to TLR4^−/−^ controls fed an SD (−24 ± 2%; Figure 6B). However, 1400 W treatment did not further change NANC-mediated relaxation in TLR4^−/−^ HFD mice (Figure 6B), suggesting that the absence of TLR4 confers partial protection against the low-grade inflammatory state induced by HFD, as previously reported [24]. In contrast, L-NAME pretreatment nearly abolished EFS-evoked NANC relaxation in ileal tissues from TLR4^−/−^ HFD mice (Figure 6B), resulting in inhibitory responses comparable to those observed in WT SD mice. Morphological analysis revealed that TLR4^−/−^ SD myenteric ganglia contained significantly fewer nNOS^+^ neurons (−26 ± 1%; Figure 6C,E) compared with WT SD mice. After HFD, the number of nNOS^+^ neurons decreased by 23% in WT myenteric ganglia (Figure 6C,E), with only minor, non-significant changes in TLR4^−/−^ LMMPs. Moreover, neuronal loss was evident exclusively in WT mice following HFD (Figure 6C,D and Supplementary Figure S2C), consistent with previous findings in both the large [13,30] and small intestine [31,32].

### 2.5. HFD Affects Enteric Neuroplasticity in a TLR4-Dependent Manner

**Figure 7:**
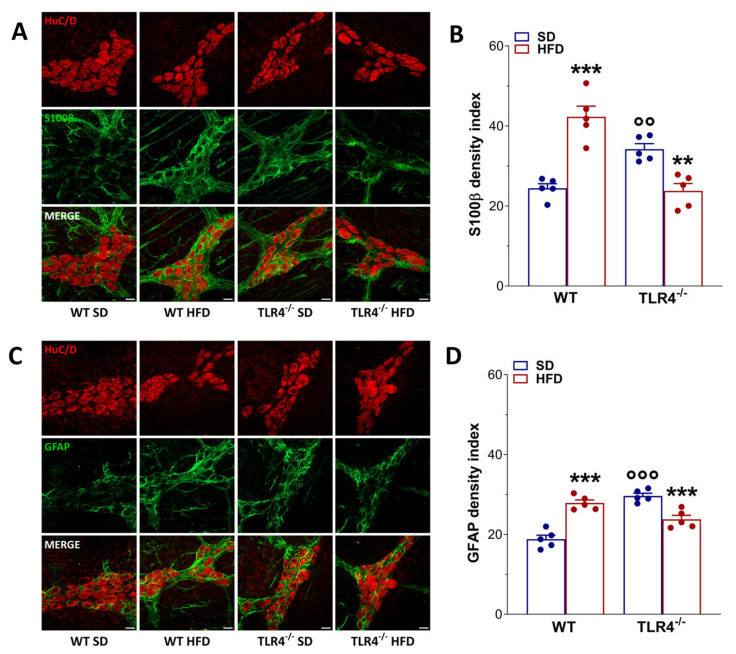
HFD-induced obesity determined neuroplastic changes in myenteric plexus architecture of WT mice. (**A**,**C**) Representative confocal microphotographs showing the distribution of HuC/D^+^ (red) neurons and S100β^+^ (green), (**A**), and glial fibrillary acidic protein (GFAP)^+^ (green), (**C**) glial cells in longitudinal muscle–myenteric plexus (LMMP) preparations obtained from WT and TLR4^−/−^ mice fed SD or HFD (scale bars = 20 μm). (**B**,**D**) Quantification of S100β (**B)** and GFAP (**D**) fluorescence intensity index; *n* = 5 mice/group. Data are reported as mean ± SEM. ** *p* < 0.01, *** *p* < 0.001 vs. related SD genotype; °° *p* < 0.01, °°° *p* < 0.001 vs. WT SD mice.

To evaluate the effect of diet on ENS structure, we quantified the number of HuC/D^+^ neurons and assessed the distribution of the glial markers S100β and GFAP. Increased immunoreactivity and expression of GFAP and S100β are well-established indicators of ENS neuroplasticity and reactive gliosis [22,23,33]. Consistent with our previous findings, loss of TLR4 signaling resulted in a significant increase in S100β and GFAP density indices, reflecting an inflammatory phenotype in EGCs when compared with WT mice fed an SD (+79 ± 2% and +15 ± 1%, respectively; Figure 7) [23]. Furthermore, TLR4^−/−^ myenteric ganglia exhibited a masked reduction in the total number of HuC/D^+^ neurons (−12 ± 1%) relative to WT SD mice (−9 ± 2%; Figure 6).

HFD administration induced reactive gliosis in WT mice, evidenced by a 2.4-fold and 1.2-fold increase in, respectively, S100β and GFAP immunoreactivity (Figure 7). In contrast, HFD appeared to normalize enteric gliosis in TLR4^−/−^ mice, reducing S100β and GFAP immunoreactivity by 25 ± 2% and 33 ± 2%, respectively (Figure 7). However, in all experimental groups, HFD led to an overall increase in the total number of SOX10^+^ cells (Supplementary Figure S2A,B). Together, these findings suggest that TLR4 signaling contributes to the development of HFD-induced small-intestine neurogliopathy and associated adaptive neuroplastic changes.

### 2.6. TLR4 Deficiency Affects 5-HTergic Neurotransmission Following HFD-Induced Obesity

Considering the impact of 8-week HFD on cholinergic- and nitrergic-mediated neuromuscular response in WT HFD mice, we next investigated the potential role of 5-HT in regulating ileal contractility in mice fed with SD or HFD. Previous studies in rodents have reported that HFD increases intestinal 5-HT levels, likely reflecting a compensatory mechanism to suppress food intake and limit weight gain [34,35].

**Figure 8:**
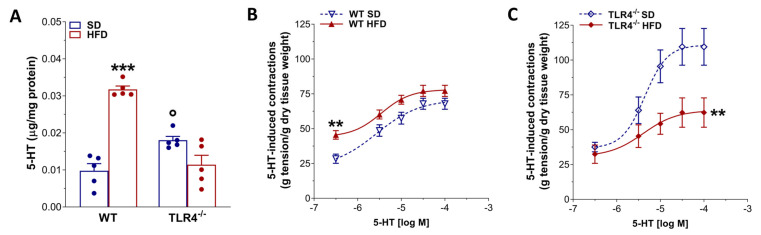
HFD-induced obesity altered 5-HT-mediated ileal contraction in TLR4^−/−^ mice. (**A**) 5-HT levels obtained by HPLC in ileal tissues of WT and TLR4^−/−^ mice fed SD or HFD; *n* = 5 mice/group. (**B**,**C**) Non-cumulative concentration-response curves to 5-HT (0.3–100 μM) in isolated ileal preparations from WT and TLR4^−/−^ animals fed SD or HFD; *n* = 10 mice/group. Data are reported as mean ± SEM. ** *p* < 0.01, *** *p* < 0.001 vs. related SD genotype; ° *p* < 0.05 vs. WT SD mice.

In our study, 5-HT levels were significantly higher in ileal tissues from TLR4^−/−^ compared with WT controls (Figure 8A). Following HFD treatment, WT mice displayed a marked increase in 5-HT levels, whereas no significant change was detected in TLR4^−/−^ animals (Figure 8A). Consistent with the known dual excitatory and inhibitory actions of 5-HT on enteric motor neurons [36], we next assessed the functional response to exogenous 5-HT. Isolated ileal segments from WT and TLR4^−/−^ mice fed an SD were incubated with increasing concentrations of 5-HT to generate non-cumulative concentration-response curves. As shown in Figure 8B, WT HFD ileal segments exhibited significantly enhanced contractile responses at 0.3 μM 5-HT, while responses at higher concentrations were comparable to those observed in SD-fed controls. In contrast, TLR4^−/−^ SD ileal segments showed an overall increase in 5-HT-induced contraction, reflected by an upward shift in the concentration–response curve (Figure 8C). HFD administration in TLR4^−/−^ mice, however, significantly reduced the 5-HT-mediated response compared with SD animals (Figure 8C).

## 3. Discussion

A diet rich in fatty food is known to determine chronic systemic mild inflammation, causing changes in gut motility, mainly constipation in humans and animal models, evidenced by slow intestinal propulsive activity [7,37]. Indeed, obesity-related low-grade systemic inflammation usually engages a complex network of signaling pathways, including those driven by gut microbiota, which may influence several organs, such as the brain, and impact behavior. However, the causal relationships linking obesity, inflammation, neurodegeneration, and metabolic disease remain not completely understood [38]. The 60% lipid content in HFD mimics a contemporary diet based on a high-fat, low-carbohydrate diet in humans, which determines body weight gain and dysmetabolic features, characteristic of moderate obesity [13]. Its consumption for more than 4 weeks showed delayed gastrointestinal transit associated with ganglionic shrinkage, reduced nerve cell soma, lower number of nitrergic neurons always in the colon, to highlight an association between gut dysmotility and enteric neuropathy [13,25,28,31,39]. However, the molecular mechanisms underlying obesity-induced colonic dysmotility and enteric neuropathy are still not fully clarified.

Although the impact of obesity on central and hormonal regulation of GI motility has been well characterized in colonic tissues, only a few studies have examined the effects of HFD and TLR4 deficiency on ENS activity of the small intestine [7,24,25,28,32]. The small intestine contains the largest population of enteric neurons in the gut and plays a pivotal role in nutrient absorption as well as in coordinating immune, neural, and endocrine responses [40]. TLR4 activity is influenced by dietary components: saturated fatty acids can activate its signaling to mediate inflammatory processes, whereas unsaturated fats may exert an opposing, anti-inflammatory effect [41]. The higher abundance of intestinal LPS-bearing bacterial species present during HFDs leads to the overactivation of TLR4 on intestinal immune and epithelial cells, contributing to the onset of inflammatory processes at the epithelial level. Furthermore, HFD-induced intestinal dysbiosis can alter intestinal barrier permeability by impairing the functions of tight junction proteins, causing the translocation of bacterial-derived molecular profiles and the possible overactivation of TLR4 expressed in other cellular subtypes (i.e., smooth muscle cells, resident macrophages, enteric neurons, and glia) within the intestinal wall [42].

Several investigations on HFD-induced obesity revealed that HFD causes impaired GI transit, increased plasma LPS concentrations, and TLR4-mediated release of pro-inflammatory cytokines and myenteric neuron apoptosis primarily in the colon [13,25].

In this study, we have characterized, for the first time, the impact of TLR4 signaling on small intestine ENS morpho-functional integrity in a mouse model of 8-week HFD-induced obesity. Specifically, the new findings of this study demonstrate that HFD-induced obesity determines in the small intestine the following effects dependent on TLR4 signaling: (i) prolonged gastrointestinal transit; (ii) complex alterations in enteric neuroglioplasticity; (iii) impaired excitatory cholinergic neuromuscular responses; (iv) reduced intestinal inhibitory motor tone, partially mediated by higher iNOS- and nNOS-derived NO signaling; (v) myenteric neurodegeneration accompanied by a marked decline in nNOS^+^ neurons; and (vi) altered 5-HT-mediated neuromuscular function associated with changes in ileal 5-HT content.

After 8 weeks of HFD, WT mice developed obesity together with significant changes in metabolic indexes (i.e., increases in blood glucose, triglycerides, and cholesterol), thus substantiating the appropriateness of this model as previously shown [6,43]. Although macroscopic assessment scores revealed no substantial differences between experimental groups, HFD feeding induced low-grade intestinal inflammation, evidenced by shortening of the gastrointestinal tract and upregulated expression of pro-inflammatory cytokines such as TNFα, IL-1β, and IL-6. Consistent with our findings, several studies have shown that HFD-fed animals show increased intestinal expression of pro-inflammatory mediators (TNF, IL-1β, IL-6), along with morphological alterations, disrupted mucin biosynthesis, and impaired mucosal barrier integrity, features that collectively underscore the central role of obesity in promoting gut inflammation and increased intestinal permeability [24,25,44].

Conversely, only after 5 weeks of HFD did TLR4^−/−^ mice reach a significantly different body weight compared to SD-fed mice, associated with changes in lipid profile and gut morphology parameters. However, HFD had no effect on glycemia and mRNA levels of mucosa-deprived pro-inflammatory cytokines, as previously shown in the colon [24]. HFD caused a marked decrease in transit time and stool water content in WT mice, with no changes in TLR4^−/−^ mice, suggesting that the absence of TLR4 signaling partially protects against the HFD-induced constipation, possibly due to changes in gut microbiota and ENS activity. TLR4 has, indeed, emerged as a critical molecular link between inflammation and insulin resistance. Beyond its classical activation by LPS, TLR4 can also be stimulated by saturated free fatty acids during the hyperlipidemic state associated with obesity and chronic HFD intake [26,45]. Mutations or deficiency of TLR4 have been shown to confer partial protection against the metabolic consequences of obesity, mitigating insulin resistance and systemic inflammation [26,41,46].

Gut inflammation is initiated when TLR4 activation, triggered by its ligands such as LPS or other pathogen-associated molecular patterns (PAMPs), leads to NF-κB activation and subsequent production of pro-inflammatory cytokines including TNFα and IL-1β [4]. Recent studies further demonstrate that inflammation alters enteric neuronal circuitry, promoting neuronal hyperexcitability, disrupting the peristaltic reflex and synaptic facilitation, and attenuating inhibitory neuromuscular transmission, changes that collectively contribute to constipation, one of the hallmark gastrointestinal manifestations of obesity [7,27].

Indeed, we identified a marked reduction in the neuromuscular excitatory cholinergic response following the 8-week HFD in WT with no difference in TLR4^−/−^ mice. These impairments in cholinergic neurotransmission could be attributed to the presence of cholinergic neural sufferance, as shown by the reduction in ChAT^+^ neurons. Since loss of ChAT^+^ neurons in proximal colon of mice fed with HFD has been observed previously [14,28,47], our findings further highlight a critical role of TLR4 signaling in neuronal plasticity, as confirmed by the immunofluorescence results.

To further characterize the impact of cholinergic dysfunction on neuromuscular contractility, we assessed NANC-mediated relaxation in isolated ileal segments from both genotypes following HFD. Under non-inflammatory conditions, WT mice exhibited an inhibitory response mediated exclusively by nNOS activity, whereas in TLR4^−/−^ mice, the inhibitory response was mediated by both iNOS and nNOS [16]. After the development of obesity, we found in WT mice a reduced inhibitory tone affected by nNOS- and iNOS-produced NO, being significantly different compared to the response in SD condition, suggesting the presence of a low-grade inflammation and loss of nNOS^+^ neurons as previously shown in the gastrointestinal tract [2,24,31,48]. TLR4^−/−^ mice fed with HFD showed no changes in the nitrergic pathways but a reduction in inhibitory response, possibly due to other inhibitory pathways [16]. No significant changes in iNOS expression have previously been reported in the colon of TLR4^−/−^ mice fed an HFD [24], further suggesting that HFD-induced alterations in gut microbiota play a pivotal role in triggering LPS-mediated intestinal inflammation and may contribute to the phenotypic features observed in HFD-fed mice.

In response to injury, stress, or inflammation, EGCs, located in the myenteric ganglia, become reactive as shown by the upregulation of their markers S100β, GFAP, and SOX10 [22,23]. Consistent with previous reports [6,10,43], we observed an increase in S100β and GFAP immunofluorescence in WT mice following HFD. Conversely, in TLR4^−/−^ myenteric ganglia, HFD determined a marked reduction in GFAP and S100β. However, in both genotypes fed with HFD, an increased number of SOX10^+^ cells was observed in the myenteric plexus. These data further support the involvement of the TLR4 pathway not only in the control of glial commitment but also in tuning adaptive neuroplastic changes and neurodegeneration, both in colon and small intestine [13,16,23,25].

The role played by 5-HT in gastrointestinal disorders remains debated, and several studies have brought forward a link connecting the serotonergic system, obesity, and inflammatory bowel disease (IBD) [34,36,49]. In the ileum of TLR4^−/−^ mice, 5-HT-evoked contractile response was significantly greater than that observed in WT mice and was accompanied by a higher 5-HT content in mucosa-deprived ileal preparations. These findings support the existence of a functional interplay between intestinal serotonergic neurotransmission and TLR4 signaling [22,50,51]. HFD did alter ileal 5-HT levels only in WT mice, indicating the presence of low-grade intestinal inflammation and impaired ileal neuromuscular function, further supporting the involvement of TLR4 signaling in obesity-associated small intestine dysmotility, consistent with previous reports [34,35].

## 4. Materials and Methods

### 4.1. Mice

Experiments were achieved using male TLR4^−/−^ (B6.B10ScN-Tlr4^lps-del^/JthJ) mice aged 9 ± 1 week old and sex- and age-matched wild-type (WT) C57BL/6J mice (Jackson Laboratories, Bar Harbor, ME, USA). All animals were housed in individually ventilated cages at the Animal Facility of the Department of Pharmaceutical and Pharmacological Sciences, University of Padova, under controlled environmental conditions (temperature 22 ± 2 °C; relative humidity 60–70%). All animals were specific pathogen-free and housed under controlled conditions with a 12/12 h light/dark cycle, receiving standard chow and tap water ad libitum. All experimental protocols were approved by the Italian Ministry of Health (authorization n° 1142/2015-PR and 624/2021-PR) and by the Animal Care and Use Ethics Committee of the University of Padova. Experiments were conducted in full compliance with the ARRIVE guidelines [52,53,54] and with national and European regulations governing the care and use of laboratory animals.

### 4.2. In Vivo Treatments

To reproduce an obese status, mice TLR4^−/−^ and WT were randomly divided into two groups and fed with a commercial standard diet (SD group; provided 3469 kcal/kg, with 10% kcal as fats, 24% kcal as proteins, and 66% kcal as carbohydrates; C 1090–10; Altromin International, Lage, Germany) with a high-fat diet (HFD group; provided 5190 kcal/g, with 60% kcal as fats, 16% kcal as proteins, and 24% kcal as carbohydrates; C 1090–60; Altromin International) [6,43]. The detailed composition of both SD and HFD is reported in the Supplementary Table S1. After 8 weeks, animals were killed by cervical dislocation. All the subsequent experimental procedures were conducted blindly.

### 4.3. Measurement of Metabolic Parameters

Blood samples were collected from the tail vein of mice fed either an SD or an HFD. After a 1 h fasting period, triglyceride, cholesterol, and glucose levels were measured using a Multicare Insensor (BSI Srl, Arezzo, Italy), according to the manufacturer’s instructions and as previously described [43,55].

### 4.4. Gastrointestinal Transit Analysis

Fluorescein isothiocyanate (FITC)-labeled dextran (70 kDa; 100 μL of 25 mg/mL in 0.9% saline) was administered by gavage to mice [16,29]. After 30 min, the time needed for the fluorescent probe to reach the maximal concentration in the small intestine in physiological conditions [22], mice were sacrificed, and the whole GI tract from stomach to distal colon was collected, measured, and placed into Krebs solution. The stomach and caecum were weighed and analyzed separately, whereas the small intestine and colon were divided into 10 and 3 equal length segments, respectively. Luminal and fecal contents were collected and centrifuged at 12,000 rpm for 10 min at 4 °C. The fluorescence intensity of FITC-dextran in each segment was then measured using a fluorimeter (Victor, PerkinElmer; Wallac Instruments, Turku, Finland) at an excitation wavelength of 485 nm and emission at 525 nm. Gastrointestinal transit was expressed as the geometric center (GC) of the fluorescent marker distribution, calculated as follows: GC = Σ (% of total fluorescence signal per segment × segment number)/100, as previously described [29,56].

### 4.5. Stool Frequency and Colonic Emptying

Fecal pellet output and water content were evaluated in non-fasted WT and TLR4^−/−^ mice fed either an SD or HFD. Fecal water content serves as an indicator of constipation, diarrhea, or malabsorption. Each animal was placed individually in a clean, transparent cage and observed for 60 min. The number of fecal pellets expelled per hour (stool frequency) was recorded as a measure of colonic motility. Pellets were then collected and weighed (wet weight), dried at 65 °C for 24 h, and reweighed (dry weight). Fecal water content was calculated from the difference between wet and dry weights and expressed as a percentage [29].

### 4.6. Ex Vivo Contractility Studies

Intestinal contractility was examined ex vivo by recording tension changes on ileal segments using the isolated organ bath technique, as previously described [22,23,29,57]. Distal ileal segments (1 cm) from each experimental group were mounted in a 10 mL organ bath containing oxygenated Krebs solution maintained at 37 °C. Mechanical activity was monitored using isometric force transducers (World Precision Instruments, Berlin, Germany) connected to a PowerLab 4/30 data acquisition system and analyzed with LabChart 8 software (ADInstruments, Besozzo, VA, Italy).

Each preparation was subjected to an initial tension of 0.5 g and allowed to equilibrate for at least 45 min to establish rhythmic spontaneous contractions. After equilibration, tissues were challenged with 1 μM carbachol (CCh) until stable responses were obtained [29]. Cholinergic-mediated contractions were evaluated by exposing ileal segments to cumulative concentrations of CCh (0.001–100 μM) to generate concentration-response curves. Neuronal-mediated contractions were elicited by electrical field stimulation (EFS, 0–40 Hz; 1 ms pulse duration; 10 s pulse-trains, 40 V) using platinum electrodes connected to an S88 stimulator (Grass Instrument, Quincy, MA, USA). Neuronal-mediated relaxations were analyzed under non-adrenergic non-cholinergic (NANC) conditions after 20 min preincubation with 1 μM atropine + 1 μM guanethidine. To assess nitrergic inhibitory neurotransmission, EFS-induced relaxation under NANC conditions was recorded in the presence of 100 μM Nω-nitro-L-arginine methyl ester hydrochloride (L-NAME, a non-selective nitric oxide [NO] synthase [NOS] inhibitor) or 10 μM 1400 W (a selective inducible NOS [iNOS] inhibitor).

Concentration–response curves to 5-HT (0.3–100 μM) were generated in a non-cumulative manner [50,58]. Contractile responses were expressed as gram tension/gram dry tissue weight, while inhibitory motor responses were quantified by calculating the area under the curve (AUC) of relaxations using the trapezoidal rule, and values were normalized to dry tissue weight [22].

### 4.7. Immunohistochemistry

#### 4.7.1. Histology

Briefly, intestine sections with a thickness of 5–10 μm were fixed overnight in 4% paraformaldehyde and processed for paraffin embedding. Slides were treated with xylene to remove the paraffin, rehydrated through an ethanol series, and pretreated in acidic citrate buffer. The primary antibody (Rabbit Anti-Human/Mouse cleaved caspase-7, Cell Signaling, Danvers, MA, USA) was diluted 1:1000 and applied overnight at 4 °C. Rabbit secondary antibody and polymer HRP (Novolink Polymer Detection Systems, Leica, UK) and chromogen substrate diaminobenzidine (Dako, Carpinteria, CA, USA) reactions were used to visualize positive cells as brown. Slides were counterstained by hematoxylin to clearly distinguish the cell nuclei [56].

#### 4.7.2. Ileal Whole Mount Preparations

To evaluate the effect of HFD on the architecture of the enteric neuroglial network, freshly isolated distal ileum segments (10 cm) were rinsed with Krebs solution to remove luminal contents and subsequently fixed in 4% paraformaldehyde (PFA) in PBS for 2 h at room temperature. After three 30 min PBS washes, the ileal segments were cut into 0.5 cm pieces, opened along the mesenteric border, and placed mucosal side down on the bottom of Sylgard-coated dishes. Using a dissecting microscope, longitudinal muscle–myenteric plexus (LMMP) whole-mount preparations were isolated as previously described [59]. LMMPs from all experimental groups were pinned flat onto Sylgard-coated dishes and washed in PBS containing 0.3% Triton X-100 (PBT) for 45 min with gentle agitation. Non-specific binding sites were blocked using 5% bovine serum albumin (BSA) in PBT for 1.5 h at room temperature, after which preparations were incubated overnight with primary antibodies (Table 1) diluted in PBT and 5% BSA. The next day, samples were washed in PBT and incubated for 2 h at room temperature with the secondary antibodies (Table 1), also diluted in PBT with 5% BSA. Following immunolabeling, LMMP preparations were then mounted on glass slides using Mowiol mounting medium (CitiFluor™ Mountant Solution AF1) and stored in the dark at −20 °C until imaging. Negative controls were prepared by either substituting primary antibodies with isotype-matched controls at equivalent concentrations or by preincubating primary antibodies with corresponding control peptides (final concentration as recommended by the manufacturer). All immunohistochemical procedures were performed in accordance with the guidelines of the British Society of Pharmacology [60].

#### 4.7.3. Confocal Image Acquisition and Analysis

Confocal imaging was performed using a Zeiss LSM 800 system (Oberkoken, Germany) equipped with 20× and 63× oil-immersion objectives (NA 1.4), as previously described [22]. Briefly, Z-stack images (25 optical planes; 512 × 512 pixels) were acquired and processed as maximum intensity projections for LMMP whole-mount preparations. All microscope settings were maintained constant across samples. In LMMPs, the total number of myenteric neurons was quantified by counting the number of HuC/D^+^ cells in 10 randomly selected images per mouse, and normalized to ganglionic area. The distribution of nitrergic neurons and glial cells was assessed by blind counting of nNOS^+^ neurons and SOX10^+^ glial cells, respectively, and normalized to the ganglionic area [16,29].

To evaluate fluorescence intensity (density index) of GFAP, S100β, and ChAT, signal intensity was measured in 10 randomly acquired images from the ileal neuromuscular compartment of each mouse (*n* = 5 mice/group), as previously described [23,29,61]. The density index for each marker was calculated as fluorescence intensity per myenteric ganglion area and was expressed as mean ± SEM. All image analyses were performed using ImageJ (Fiji), version number 1.54i.

### 4.8. RNA Isolation and Quantitative RT-PCR

Total RNA was extracted from mucosa-deprived small intestine samples using TRIzol (Invitrogen, Carlsbad, CA, USA) and treated with DNase I (DNase Free, Ambion, ThermoFisher, Monza, Italy) to eliminate residual genomic DNA. Two μg of total RNA were reverse-transcribed using the High-Capacity cDNA synthesis kit (Applied Biosystems, Life Technologies, Grand Island, NY, USA). Quantitative RT-PCR was performed with a QuantStudio 3 Real-Time PCR System (Thermo Fisher Scientific, Carlsbad, CA, USA) using Power SYBR Green Universal PCR Master Mix (Applied Biosystems, Foster City, CA, USA), according to the manufacturer’s protocol. Primers were designed using Primer Express software version 3.0.1 (Applied Biosystems, Foster City, CA, USA) with similar amplicon size and similar amplification efficiencies, suitable for analysis by the 2^−ΔΔCt^ method [61]. The following primer sequences were used: IL-6 (F 5′-TAGTCCTTCCTACCCCAATTTCC-3′; R 5′-TTGGTCCTTAGCCACTCCTTC-3′); IL-1β (F 5′-GCAACTGTTCCTGAACTCAACT-3′; R 5′-ATCTTTTGGGGTCCGTCAACT-3′); TNFα (F 5′-CCCTCACACTCAGATCATCTTCT-3′; R 5′-GCTACGACGTGGGCTACAG-3′). Each reaction contained 500 nM of each primer, and experiments were performed on at least seven biological replicates for the experimental group (*n* = 5), as previously described [61]. The 2^−ΔΔCt^ values, obtained by comparing normalized Ct values of HFD-treated samples with those of SHAM controls, were used to determine the effects of HFD-induced obesity on pro-inflammatory cytokine expression.

### 4.9. Quantification of Cytokines in Ileum Tissue with Enzyme-Linked Immunosorbent Assay

Mucosa-deprived small intestine samples (70 mg) were homogenized in 700 μL phosphate buffer containing 0.05% Tween-20 and phosphatase inhibitor cocktail (1:10) (PhosSTOP, Roche, Millipore Sigma, Merk Life Science S.r.l., Milan, Italy) using a tissue homogenizer (OMNI International, Merk Life Science S.r.l., Milan, Italy). Tissue homogenates were centrifuged for 15 min at 13,000 g, 4 °C, and supernatants were collected. The protein concentrations of the supernatant were determined by using the BCA Protein Assay Kit (ThermoFisherScientific) according to the manufacturer’s instructions. The concentrations of TNF-α, IL-6, and IL-1β were measured with specific ELISA kits (Mouse TNF-alpha ELISA Kit–Quantikine, cat# MTA00B-1; Mouse IL-6 Quantikine ELISA Kit, cat# M6000B-1; Mouse IL-1 beta/IL-1F2 ELISA Kit–Quantikine Kit, cat# MLB00C-1, R&D System, Bio-Techne SRL, Milan, Italy). Absorbance values from each ELISA are expressed in units of pg/mg of protein.

### 4.10. High-Performance Liquid Chromatography (HPLC) Analysis of 5-HT Levels

Ileal 5-HT levels were quantified in tissue homogenates by HPLC, as previously described [22]. Briefly, freshly isolated ileal segments were rapidly frozen in liquid nitrogen and pulverized in a chilled stainless-steel mortar containing 0.5 mL of 1 N HClO_4_. The resulting homogenates were sonicated using an Elmasonic S30 sonicator (Elma, Singer, Germany) and centrifuged at 13,000× *g* for 30 min at 4 °C. The supernatants were stored at −80 °C until HPLC analysis, while the pellets were resuspended in 1 N NaOH, boiled for 20 min at 60 °C, and then centrifuged at 15,000× *g* for 10 min at 4°C for protein determination.

Prior to analysis, the supernatants were adjusted to pH 4–5 with 1 N NaOH and analyzed using a Shimadzu LC-10AD HPLC system (Shimadzu, Milan, Italy) equipped with a fluorometric detector (Shimadzu RF-10AXL; excitation 285 nm, emission 345 nm). Chromatographic separation of tryptophan metabolites was achieved on an Apollo EPS C18 100A column (5 μm; 250 × 4.6 mm; Grace, Deerfield, IL, USA) with an Alltech guard column containing RP-8 stationary-phase (25–40 μm LiChroprep; Merk Life Science S.r.l., Milan, Italy). Kynurenine analysis was performed on a Grace Smart RP-18 column (5 μm; 250 × 4.6 mm; Grace) using a UV–Vis detector (SPD-10A, Shimadzu, Milan, Italy) set at 360 nm. The mobile phases consisted of the following: Phase A, 95% acetonitrile/5% water, and Phase B, 90% water/5% methanol (pH 3.8). Analytes were eluted under isocratic conditions (5% Phase A, 95% Phase B, *v*/*v*) at a flow rate of 1 mL min^−1^.

### 4.11. Chemicals

Unless otherwise specified, all chemicals were obtained from Sigma–Aldrich (Milan, Italy) and were of the highest commercially available analytical grade. PFA and mounting solution (Citifluor AF1) were purchased from Electron Microscopy Sciences-Società Italiana Chimici (Rome, Italy), and Triton-X-100 was obtained from Applichem (Milan, Italy). All drugs for in vitro contractility studies were dissolved in Milli-Q water.

### 4.12. Statistical Analysis

All data are presented as mean ± SEM, except for geometric center values, which are reported as median (range, minimum–maximum). All analyses were performed by investigators blinded to treatments using GraphPad Prism software (version 8.4; San Diego, CA, USA). Animals were randomly assigned to four experimental groups. Data distribution was assessed using the Shapiro–Wilk normality test. Statistical significance was determined using the paired or unpaired Student’s *t*-test for two-sample comparisons, two-way ANOVA followed by the Bonferroni post hoc test for multiple comparisons, or the nonparametric Mann–Whitney U test for independent variables.

Differences were considered significant at *p* < 0.05. ‘N’ indicates the number of animals per group. Post hoc analyses were conducted only when the ANOVA F-test reached significance (*p* < 0.05) and variance inhomogeneity was confirmed.

All data handling and statistical procedures adhere to the current recommendations for experimental design and analysis in pharmacology [52,54].

## 5. Conclusions

The intricate pathogenesis of enteric neuropathy in obesity arises from the interplay of multiple factors, which must be identified and evaluated as potential pharmacological targets. Overall, our findings underscore the critical impact of TLR4 signaling in mediating the deleterious consequences of an HFD on the morpho-functional integrity of the ENS. Further research is warranted to determine the contribution of dietary-associated molecular patterns and/or microbiota-associated molecular profiles associated with HFD-induced intestinal dysbiosis in driving the morphofunctional changes in the ENS during HFD. A more comprehensive understanding of the microbiota–neuroimmune interactions within the gut will be the key to developing more effective therapeutic strategies for obesity-associated intestinal disorders and facilitating the translation of preclinical findings into clinical applications.

## Figures and Tables

**Table 1:** Primary and secondary antibodies and their respective dilutions used for immunohistochemistry on ileal whole-mount preparations.

Antibody	Host Species	Dilution	Catalog Number	Source
Primary Antisera (Clone)				
HuC/D (monoclonal)	Mouse biotin-conjugated	1:100	A-21272	Thermo Fisher Scientific (Monza, Italy)
nNOS (polyclonal)	Rabbit	1:100	61–700	Thermo Fisher Scientific
GFAP (polyclonal)	Chicken	1:100	ab4674	Abcam (Cambridge, UK)
S100β (polyclonal)	Guinea pig	1:100	ab10353	Abcam
SOX10 (monoclonal)	Rabbit	1:50	ab155279	Abcam
ChAT (polyclonal)	Goat	1:50	AB144P	Sigma-Aldrich (Milan, Italy)
Secondary Antisera				
Donkey anti-goat IgY Alexa 555-conjugated	-	1:500	A-21432	Thermo Fisher Scientific
Streptavidin Alexa 555-conjugated	-	1:1000	S21381	Thermo Fisher Scientific
Streptavidin Alexa 488-conjugated	-	1:1000	S11223	Thermo Fisher Scientific
Goat anti-Chicken IgY Alexa 488 conjugated	-	1:1000	A-11039	Thermo Fisher Scientific
Goat anti-Guinea Pig IgG Alexa 488 conjugated	-	1:1000	A-11073	Thermo Fisher Scientific
Goat anti-Rabbit IgG Alexa 488-conjugated	-	1:1000	A-11008	Thermo Fisher Scientific

## Data Availability

The original contributions presented in this study are included in the Supplementary Materials. Further inquiries can be directed to the corresponding authors.

## Supplementary Materials

The supporting information can be downloaded at: https://www.mdpi.com/article/10.3390/ijms262110710/s1.

## Abbreviations

The following abbreviations are used in this manuscript:

5-HT	Serotonin
CCh	Carbachol
ChAT	Choline acetyltransferase
EFS	Electric field stimulation
EGCs	Enteric glial cells
ENS	Enteric nervous system
GFAP	Glial fibrillary acidic protein
HFD	High-fat diet
iNOS	Inducible NOS
LMMP	Longitudinal muscle–myenteric plexus
nNOS	Neuronal NOS
SD	Standard diet
TLRs	Toll-like receptors
