# Supplementary material for: Small Intestine Neuromuscular Dysfunctions and Neurogliopathy in a Mouse Model of High-Fat Diet-Induced Obesity: Involvement of Toll-Like Receptor 4
Source: Int J Mol Sci. 2025 Nov 3;26(21):10710. doi: 10.3390/ijms262110710 (PMC12608152; doi:10.3390/ijms262110710)
Supplement: Supplementary file 1 [file ijms-26-10710-s001.zip › ijms-3749949-supplementary.pdf]

Faggin et al., 2025 - *Manuscript ijms-3749949*

Supplemetary Figures and Table

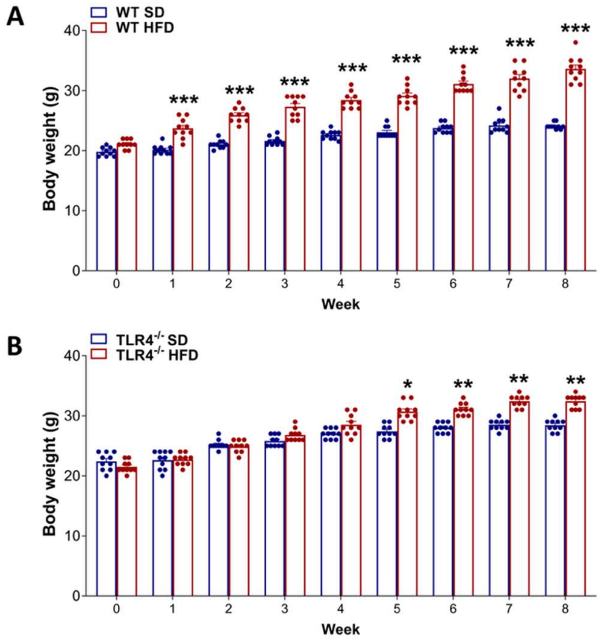

Supplementary Figure 1: TLR4 signaling partially protects against HFD-induced obesity. Bar chart of body weight gain/week in WT (**A**) and TLR4$^{-/-}$ (**B**) mice during the 8-week HFD treatment; N=10 mice/group. *P<0.05, **P<0.01, ***P<0.001 vs related SD genotype.

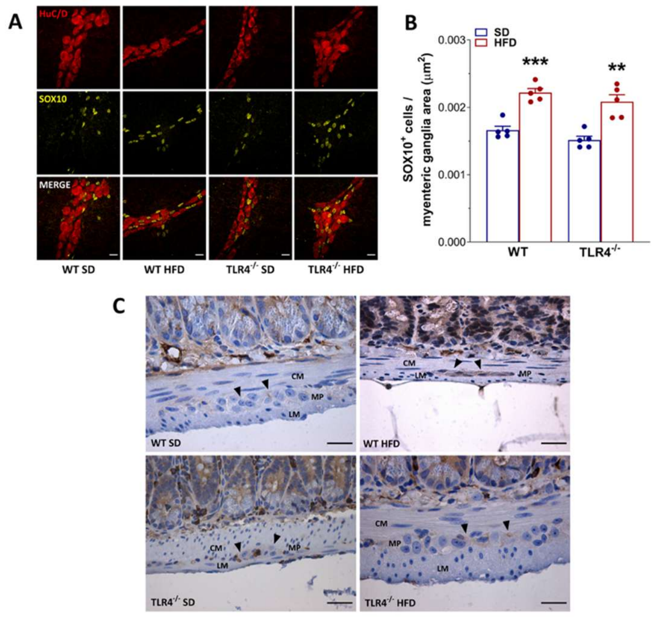

Supplementary Figure 2: TLR4 involvement in HFD-induced enteric neurogliopathy A) Representative confocal microphotographs showing the distribution of HuC/D (red) and SOX10 (yellow) and **B)** analysis of SOX10^{+} cells in ileal LMMPs of WT and TLR4$^{-/-}$ mice fed with SD or HFD (scale bars = 20 µm). Data are reported as mean ± SEM. **P<0.01 , ***P<0.001 vs related SD genotype. C) Cleaved caspase-7 immunostaining in ileal neuromuscular sections of WT and TLR4$^{-/-}$ mice fed with SD or HFD (scale bars = 100 µm). In myenteric ganglia cleaved caspase-7^{+} neurons are marked with a black arrowhead. LM=longitudinal muscle; MP=myenteric plexus; CM=circular muscle.

Supplementary Table 1: Detailed composition of the standard diet (SD; C 1090 - 10, Altromin International) and high fat diet (HFD; C 1090 – 60, Altromin International) used in this study.

Nutrients	SD (3,469 kcal/kg)	HFD (5,190 kcal/kg)
**Carbohydrates**	[%]	[%]
Monosaccharides	2	10
Disaccharides	12	3
Polysaccharides	43	16
**Minerals**	[%]	[%]
Calcium	0.7	0.6
Potassium	0.5	0.4
Magnesium	0.06	0.05
Sodium	0.4	0.4
Phosphorus	0.6	0.5
**Fatty acid**	[mg/kg]	[mg/kg]
Arachidic acid C-20:0	93	1,008
Eicosanoic acid C-20:1	56	560
α-Linolenic acid C-18:3	512	5,880
Linolenic acid C-18:2	4,236	44,520
Palmitic acid C-16:0	3,396	38,360
Stearic acid C-18:0	1,944	22,400
Oleic acid C-18:1	2,636	28,560
**Amino acids**	[mg/kg]	[mg/kg]
Alanine	9,951	9,894
Arginine	14,189	14,227
Aspartic acid	24,790	24,886
Cystine	3,455	3,448
Glutaminc acid	51,525	51,637
Glycine	8,267	8,268
Histidine	5,743	5,742
Isoleucine	10,534	10,557
Leucine	12,320	12,209
Lysine	6,654	6,659
Methionine	3,452	3,447
Phenylalanine	7,836	7,815
Proline	9,755	9,673
Serine	10,565	10,565
Threonine	8,262	8,266
Tryptophan	3,420	3,439
Tyrosine	7,594	7,583
Valine	5,561	5,524
**Trace elements**	[mg/kg]	[mg/kg]
Aluminium	9.45	7.48
Chlorine	2,420.00	2,117.50
Iron	151.13	135.35
Flourine	2.78	2.43
Iodine	0.30	0.26

Cobalt	0.11	0.08
Copper	7.12	6.56
Manganese	69.38	60.81
Molybdenum	0.13	0.12
Sulfur	2,470.87	2,365.15
Selenium	0.17	0.14
Zinc	24.82	22.31
Added vitamins	per kg	per kg
Biotin	200 μg	200 μg
Choline chloride	1,000 mg	1,000 mg
Folic acid	10 mg	10 mg
Nicotinic acid	50 mg	50 mg
Pantothenic acid	50 mg	50 mg
Vitamin A	15,000 IU	15,000 IU
Vitamin B1	20 mg	20 mg
Vitamin B2	20 mg	20 mg
Vitamin B6	15 mg	15 mg
Vitamin B12	30 μg	30 μg
Vitamin C	20 mg	20 mg
Vitamin D3	500 IU	500 IU
Vitamin E	150 mg	150 mg
Vitamin K3	10 mg	10 mg
